# Determinants of inappropriate setting allocation in the care of patients with type 2 diabetes: A population-based study in Reggio Emilia province

**DOI:** 10.1371/journal.pone.0219965

**Published:** 2019-07-22

**Authors:** Paola Ballotari, Francesco Venturelli, Valeria Manicardi, Massimo Vicentini, Francesca Ferrari, Marina Greci, Mariarosa Maiorana, Paolo Giorgi Rossi

**Affiliations:** 1 Epidemiology Unit, Azienda USL-IRCCS di Reggio Emilia, Reggio Emilia, Italy; 2 Clinical and Experimental Medicine PhD Program, University of Modena and Reggio Emilia, Modena, Italy; 3 Diabetes Clinic, Azienda USL-IRCCS di Reggio Emilia, Reggio Emilia, Italy; 4 Department of Primary Care, Azienda USL-IRCCS di Reggio Emilia, Reggio Emilia, Italy; 5 Nephrology Unit, Azienda USL-IRCCS di Reggio Emilia, Reggio Emilia, Italy; Mahidol University, THAILAND

## Abstract

The study aims to describe the distribution of patients with type 2 diabetes (T2D) by care plan and to highlight determinants of underuse and overuse of integrated care (IC). This cross-sectional study included all T2D patients resident in Reggio Emilia on 31/12/2015 based on the population-based diabetes registry. Eligibility for IC requires good glycaemic control, no rapid insulin, no kidney failure and no diabetes complications. We calculated the proportion of IC underuse and overuse and adjusted prevalence estimate using multivariate logistic regression. Determinants were age, sex, citizenship, district of residence and time since diagnosis. Of 29,776 patients, 15,364 (51.6%) were in diabetes clinic plan, 9851 (33.1%) in IC plan and 4561 (15.3%) not in any care plan (i.e., in Other group). There were 10,906 (36.6%) patients eligible for IC, of whom 1000 in Other group. When we adjusted for all covariates and restricted the analysis to patients included in care plans, the proportion of those eligible for IC plan but cared for in diabetes clinic plan (i.e. underuse of IC) was 28% (n = 3028/9906; 95%CI 27–29). Similarly, the proportion of those not eligible for IC but cared for in IC plan (i.e. overuse of IC) was 11% (n = 1720/11,896; 95%CI 10–11).The main determinant of both IC underuse and overuse was the district of residence. Foreign status was associated with underuse (37%; 95%CI 33–43), while old age (≥80 years) with both underuse (36%; 95%CI 0.33–0.38) and overuse (23%; 95%CI 22–25). The criterion for suspension of IC plan most frequently found was renal failure, followed by hospitalization for diabetes-related complications. Patients are more often allocated to more specialized settings than not. Healthcare provider-related factors are the main determinants of inappropriate setting allocation.

## Introduction

Diabetes has reached pandemic proportions, affecting almost 415 million people globally in 2015, with an increasing prevalence trend [1,2]. Type 2 diabetes (T2D) is a multifactorial chronic disease and represents one of the main risk factors for cardiovascular diseases, the leading cause of death and disability in developed countries [3,4]. Moreover, diabetes impairs quality of life and psychological well-being, including in terms of disability-adjusted life years (DALY) [4–6]. Indeed, the burden of diabetes is significant in terms of morbidity and mortality, but also has a relevant impact on socioeconomic and healthcare-related costs, especially due to diabetes-related long-term complications [7].

In the last decades, diabetic outpatient clinics (DC) have been set up in many countries, including in Italy [8–11], where usual care of T2D requires a referral by a general practitioner (GP) to a DC for diagnostic confirmation, treatment, prevention and early diagnosis of complications through close patient follow up by a team of diabetologists, nurses and dieticians, and for scheduling of regular follow up visits. This model of care is quite intensive and resource-consuming, and diabetic clinics have limited capabilities. Moreover, not all the phases of care require the same level of expertise or have the same level of complexity [12]. Recently, many healthcare systems have adopted a model of integrated care involving specialists in the most critical phases and for more complicated patients, while GPs take care of the routine management of the disease in less complex cases [9,13,14]. In this context, evidence on the effectiveness of shared care is growing [15,16].

In Italy, the 2010 Italian Standards for the Treatment of Diabetes Mellitus recommended the adoption of an integrated care model for patients with uncomplicated diabetes [17].

The Local Health Authority (LHA) in the Reggio Emilia province gradually introduced the integrated care (IC) plan starting in 2004 as a pilot project involving both diabetes clinics and GPs and targeting the T2D patients at low risk of complications [18]. Implementation took place over time and across GPs due to organizational and logistical factors and to GPs’ and patients’ attitudes (participation in IC is voluntary for both patients and GPs). This pilot project was consistent with the national project IGEA, a comprehensive strategy for implementing a chronic disease management intervention for people with diabetes, integrating GPs and specialized clinics in the management of diabetes [19]. The Emilia-Romagna health authority scaled up the IC project region-wide, issuing guidelines in 2009 that defined patients eligible for integrated management of care [18], subsequently updated [20,21].

Although the eligibility criteria for IC have been defined, the accuracy in allocating T2D patients to the correct care plan can be difficult. Indeed, inaccurate evaluation of eligibility criteria or professionals’ or patients’ unwillingness to engage with IC may cause inappropriate management: complicated patients may be allocated to a care plan that does not satisfy the clinical complexities of their needs, while patients without complications may be allocated to a care plan they do not really need. Finally, there are those patients who are not cared for in any care plan.

The present study aims to describe the current distribution of T2D patients by care plan and to highlight the main predictors of under- and overuse of integrated care plan.

## Materials and methods

### Study population and study design

Reggio Emilia province is situated in northern Italy and has approximatively 530,000 inhabitants, 300 general practitioners, six outpatient diabetes clinics and one diabetes unit in the main hospital.

In this cross-sectional study we analyzed the allocation by care plan of T2D patients prevalent on 31/12/2015, then we classified them based on eligibility to IC to measure the proportion of T2D patients not appropriately allocated. Finally, we described the determinants of inappropriate allocation: overuse of integrated care plan, (i.e., ineligible patients allocated to integrated care plan); underuse of integrated care plan, (i.e., eligible patients allocated to diabetes clinic plan).

### Description of the care model

Three groups of T2D patients were created: 1) exclusively cared for by DC from initial diagnostic assessment to periodic examinations and follow-up visits; 2) cared for both by GP and diabetes clinic through an IC plan envisaging an initial assessment by DC and a quarterly follow-up visit, one every two years at the DC, the others performed by GP; 3) Other-group (neither DC nor IC), perhaps only cared for by own GP but also voluntary opt-outs who turn to private care and neglected patients.

### Eligibility to IC criteria

**Fig 1:**
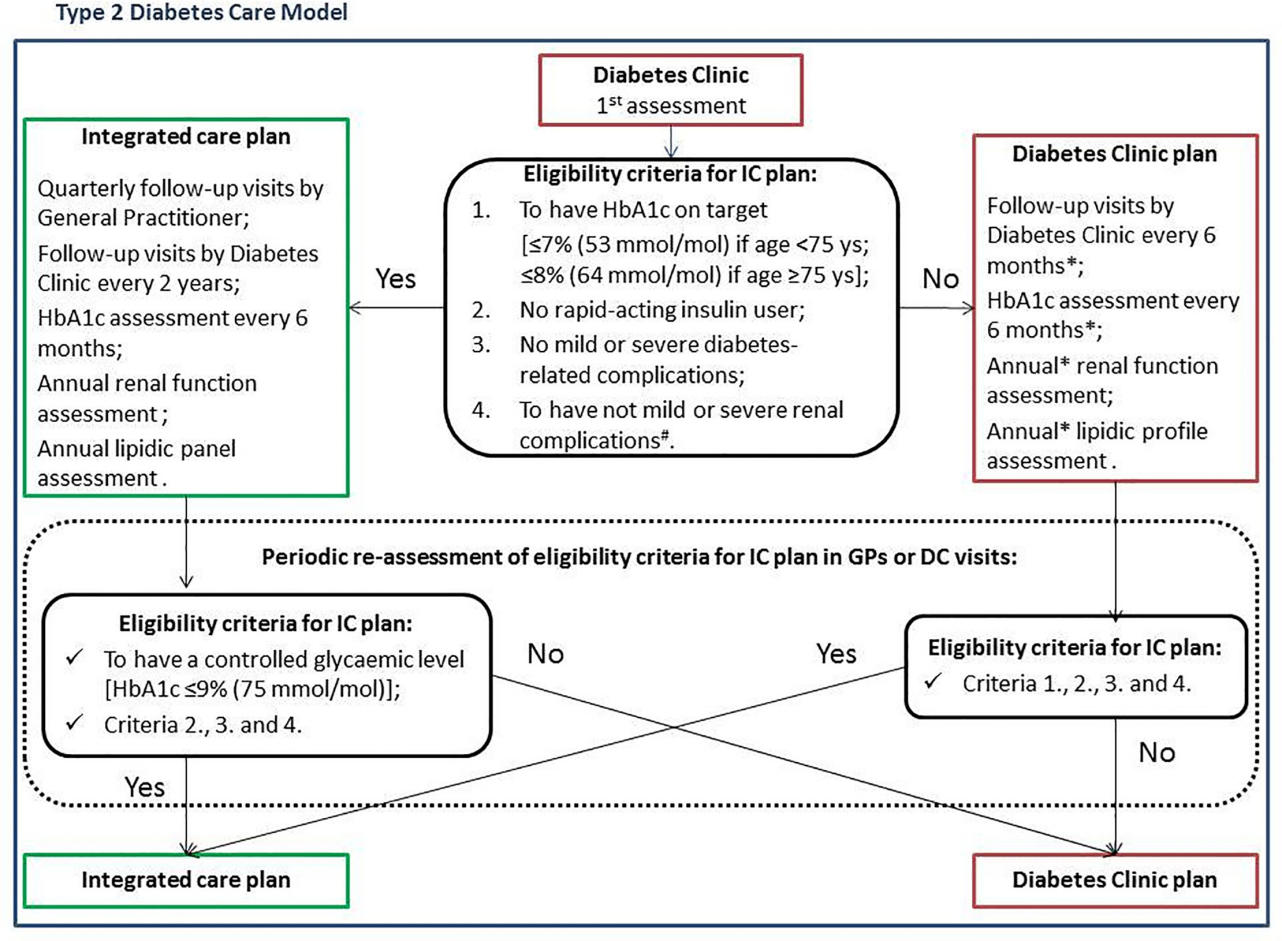
Integrated care model. Structure of the integrated care model and eligibility criteria to integrated care (IC) plan, defined according to regional guidelines criteria [18]. *Or with different time interval according to clinical conditions; ^#^Glomerular Filtration Rate level ≥60ml/min/1.73m^2^.

Fig 1 shows the structure of the integrated care model and the eligibility criteria to IC plan, defined according to regional guidelines criteria [18] (Fig 1). The algorithms used to assess eligibility criteria to the IC plan from routinely-collected data are detailed in S1 and S2 Tables. In brief, the required criteria are good glycaemic control, no rapid insulin, no kidney failure, and no diabetes complications. Eligibility is assessed at the first visit at the diabetes clinic and re-assessed during the follow-up visits. In the first visit, the unmet criteria induce the application of the DC plan until the next scheduled visit; in the follow-up visit, if at least one criterion is unmet, a suspension of the IC plan should be generated.

### Outcomes, covariates and data sources

Outcomes were “care plan in use” and “eligibility to IC” (yes or no) for all T2D patients alive and resident in Reggio Emilia province on December 31, 2015. Covariates were sex, age, citizenship, district of residence and time since diabetes diagnosis. Demographic variables were retrieved from civil registry while diabetes information was retrieved from the Reggio Emilia Diabetes Registry (REDR), a validated registry created by the deterministic linkage of six routinely collected data sources through a definite algorithm able to ascertain cases and to distinguish the type of diabetes and model of care [22]. Data have been included since 2009, and the REDR is updated annually. The date of inclusion in the registry is the date when a person first meets one of the following inclusion criteria: (1) Disease-specific exemption database: exemption from co-payment due to diabetes; (2) Hospital discharge database: hospitalisation with diabetes diagnosis in whichever position by ICD-9 (International Classification of Diseases Clinical Modification, 9th Edition) codes 250.xx; 357.2x; 362.0x; 366.41; 648.0x, excluding MDC14; (3) Biochemistry laboratory database: two glycated haemoglobin (HbA1c) test > = 6.5% (48 mmol/mol) or one HbA1c> = 6.5% and (48 mmol/mol) followed by one fasting blood sugar test> = 126 mg/dl; (4) Drug prescription databases: at least two prescriptions of antidiabetic drugs in pharmacy distribution database or only one included in direct-distribution database; (5) Diabetes outpatient clinic database: diagnosis by a diabetologist; (6) Mortality registry: cause of death by ICD-10 (International Classification of Diseases, 10th Edition) codes E10 –E14. Women with gestational diabetes or women receiving treatment for a polycystic ovarian syndrome or obesity were excluded. Cases initially notified to the registry through record linkage were retained in case they were clinically confirmed by a diabetologist or another physician.

### Statistical methods

We compared patients' baseline characteristics stratified by current care plan (i.e. DC, IC and other-group) and then we classified patients accordingly to appropriate allocation. We calculated the average predicted probabilities only for patients cared for by a care plan (i.e. IC or DC), applying a multivariate logistic model and using remaining covariates at their mean values. The covariates were sex, age, district, time since diagnosis and citizenship. Allocation to the correct care plan was used as a reference. Moreover, we calculated the intraclass correlation (ICC) by using a three-level logistic model with random effects at the district and GP levels. Finally, we described the distribution of ineligible patients by a number of met suspension criteria, and we computed the probability of each suspension criterion. The analyses were performed using the STATA statistical package Version 13.0.

### Ethical approval

This is an observational study, and the data were collected retrospectively. The Local Health Authority of Reggio Emilia was responsible for collecting and processing the data. The analyses here presented are part of a larger study named "Reggio Emilia Diabetes Registry" and has been approved by the provincial Ethics Committee on July 23, 2014 (Comitato Etico Provinciale of Reggio Emilia, now Comitato Etico AVEN, after merging with the other Ethics Committees of the Modena, Piacenza and Parma provinces http://www.aou.mo.it/ComitatoEticoAVEN). In accordance with the Italian privacy law, no patient or parental consent is required for large retrospective population-based studies approved by the competent Ethics Committee if data are published only in aggregated form.

## Results

Based on REDR, as of December 31, 2015 the resident population with DM2 was 29,776 (5.5% crude prevalence), of whom 9851 (33.1%) in IC plan, 15,364 (51.6%) cared for exclusively in DC and 4561(15.3%) not included in any defined care plan. Those in the IC plan were more likely to be aged 60–80 and with a more recent diagnosis than those cared for in DC. Relevant differences in the proportions of patients in the IC plan were appreciable between districts, and foreigners were more often cared for in DC. There were slightly more females among those without any defined care plan, as there were patients over age 80 and foreigners, while the newly diagnosed were appreciably more represented (Table 1).

**Table 1:** Characteristics of the study sample.

			Distribution by care plan						
	Total		IC		DC		Other		
Subgroup	N	%	N	%	N	%	N	%	P^
**N**	29776		9851		15364		4561		
**Sex (N;%)**									<0.0001
Females	13400	45.0	4434	45.0	6679	43.5	2287	50.1	
Males	16376	55.0	5417	55.0	8685	56.5	2274	49.9	
**Age (N;%)**									<0.0001
<60 yr	6781	22.8	1847	18.7	3961	25.8	973	21.3	
60–69 yr	7627	25.6	2736	27.8	3913	25.5	978	21.4	
70–79 yr	8623	29.0	3166	32.1	4190	27.3	1267	27.8	
80+ yr	6745	22.7	2102	21.3	3300	21.5	1343	29.4	
**District of residence (N;%)**									<0.0001
Mountain	2197	7.4	705	7.2	1166	7.6	326	7.1	
Eastern plain	3250	10.9	1737	17.6	1262	8.2	251	5.5	
Northern plain	4530	15.2	1794	18.2	2237	14.6	499	10.9	
Western hills	3770	12.7	1125	11.4	2003	13.0	642	14.1	
Capital	11773	39.5	2918	29.6	6868	44.7	1987	43.6	
Eastern hills	4286	14.4	1572	16.0	1858	12.1	856	18.8	
**Time since diagnosis (N;%)**									<0.0001
<5 yr	8986	30.2	2993	30.4	3517	22.9	2476	54.3	
5–9 yr	10594	35.6	4239	43.0	4624	30.1	1731	38.0	
10+	10196	34.2	2619	26.6	7223	47.0	354	7.8	
**Citizenship (N;%)**									
Italians	27561	92.6	9406	95.5	13968	90.9	4187	91.8	<0.0001
Foreigners	2215	7.4	445	4.5	1396	9.1	374	8.2	

Current distribution of type 2 diabetes status by care plan (2015).

IC = integrated care plan; DC = diabetes clinic care plan; other = neither IC nor DC

^ p-value of the difference among care plans

**Fig 2:**
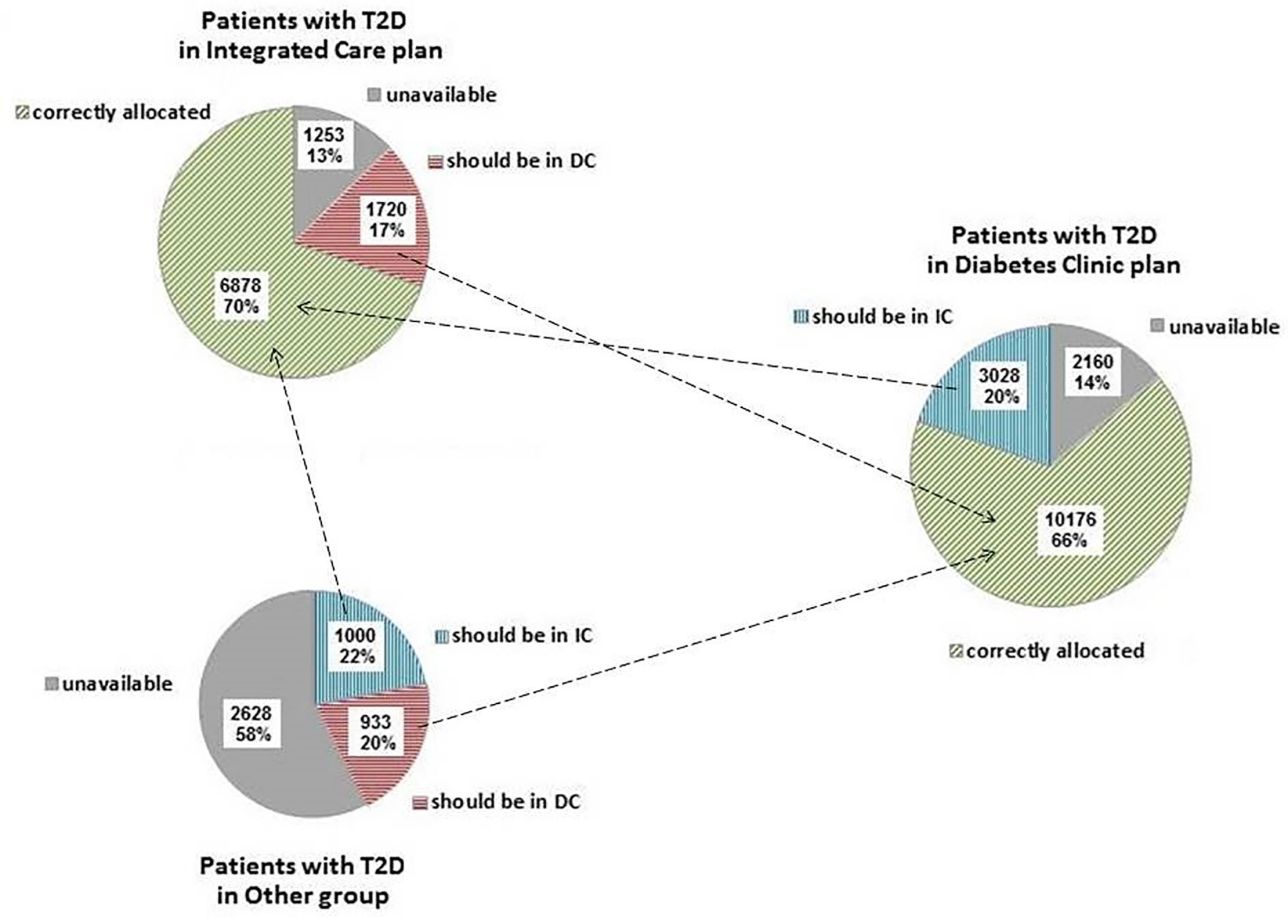
Distribution by care plan. Distribution of patients by eligibility criteria in different care plans. Unclassifiable = patients without the required laboratory assessment (i.e., without a measure of HbA1c in 2015 and/or without a measure of glomerular filtration rate in 2014–2015); Should be in IC plan = patients satisfying eligibility criteria; Should be in DC plan = patients with last 2015 HbA1c level> = 9% (75 mmol/mol) and/or at least one hospitalisation in 2012–2014 for diabetes-related causes and/or rapid-acting insulin user in 2015 and/or with last 2014–2015 GFR level <60 ml/min/1.73m^2^). IC = integrated care plan, DC = diabetes clinic plan.

Based on eligibility criteria, 10,906 (36.6%) T2D patients were eligible for IC plan and 6041 (20.3%) were not classifiable because the information was missing on some eligibility criteria. Among the eligible patients, 6878 (63.1%) were correctly allocated to the IC plan. On the other hand, 1720 patients in the IC plan should have been in the DC plan because at least one of the suspension criteria was met, and 1253 was not classifiable. Among those in the DC plan, 3028 (20%) were eligible for IC plan, and 2160 were not classifiable. Finally, among those without any defined care pathway, 2628 (58%) were not classifiable and the rest were almost equally eligible and ineligible for IC plan (Fig 2).

When adjusting for all covariates, 28% of T2D patients eligible for IC plan were followed in the DC plan (IC underuse), while 11% of ineligible T2D patients were cared for in the IC plan (IC overuse) (Table 2).

**Table 2:** Probability of inappropriate care plan assignment.

	Eligible to IC plan					Ineligible to IC plan				
Subgroup	Total	in IC	In DC	adjusted*		Total	in DC	in IC	adjusted*	
				P (unfit)	95%CI				P(unfit)	95%CI
N	9906	*6878*	*3028*	0.28	0.27–0.29	11896	*10176*	*1720*	0.11	0.10–0.11
**Sex**										
Females	4371	*3086*	*1285*	0.27	0.26–0.28	5304	*4462*	*842*	0.11	0.10–0.12
Males	5535	*3792*	*1743*	0.29	0.28–0.30	6592	*5714*	*878*	0.11	0.10–0.12
**Age**										
<60 yr	2090	*1361*	*729*	0.33	0.30–0.35	2558	*2416*	*142*	0.04	0.03–0.05
60–69 yr	2779	*2117*	*662*	0.22	0.21–0.24	3027	*2762*	*265*	0.07	0.06–0.08
70–79 yr	3200	*2253*	*947*	0.27	0.26–0.29	3399	*2820*	*579*	0.15	0.14–0.17
80+ yr	1837	*1147*	*690*	0.35	0.33–0.38	2912	*2178*	*734*	0.23	0.22–0.25
**District**										
Mountain	805	*500*	*305*	0.37	0.34–0.41	897	*726*	*171*	0.14	0.12–0.17
Eastern plain	1508	*1351*	*157*	0.10	0.08–0.11	1232	*937*	*295*	0.20	0.18–0.22
Northern plain	1535	*1196*	*339*	0.21	0.19–0.23	1862	*1532*	*330*	0.15	0.13–0.16
Western hills	1197	*807*	*390*	0.32	0.30–0.35	1554	*1376*	*178*	0.09	0.08–0.10
Capital	3425	*1930*	*1495*	0.44	0.42–0.45	4815	*4381*	*434*	0.07	0.06–0.08
Eastern hills	1436	*1094*	*342*	0.24	0.22–0.27	1536	*1224*	*312*	0.17	0.15–0.19
**Time since diagnosis**										
<5 yr	3268	*2192*	*1076*	0.31	0.29–0.33	2175	*1817*	*358*	0.17	0.15–0.19
5–9 yr	3958	*2961*	*997*	0.23	0.21–0.24	3549	*2808*	*741*	0.18	0.17–0.20
10+	2680	*1725*	*955*	0.34	0.32–0.36	6172	*5551*	*621*	0.07	0.06–0.07
**Citizenship**										
Italians	9419	*6579*	*2840*	0.28	0.27–0.29	11023	*9341*	*1682*	0.11	0.11–0.12
Foreigners	487	*299*	*188*	0.38	0.33–0.43	873	*835*	*38*	0.05	0.04–0.07

Probability of inappropriate care plan assignment for patients with type 2 diabetes by patient characteristics. Eligible to IC = includes patients eligible to IC cared in DC plan and patients in IC plan without suspension criteria. Ineligible to IC = includes patients not eligible to IC cared in DC plan and patients in IC plan with at least one suspension criteria *Adjusted P (unfit): average predicted probabilities of inappropriate care plan assignment, obtained using a logistic multivariate model and using remaining covariates at their mean values. The covariates were sex, age, district, time since diagnosis and citizenship. Placement in the correct care setting was used as a reference.

The strongest predictor of IC underuse was the district of residence; the same was true for IC overuse. In most cases, in districts where the proportion of underuse was lower than the provincial average, the proportion of overuse was also higher than average, the only exception being the mountain district, where both were above the average. No difference between males and females was present, while older age was a determinant of both over- and underuse, and people below age 60 had higher underuse and lower overuse. Small differences were also observed in terms of the duration of disease: recently diagnosed patients had slightly higher over- and underuse, while patients who had been diagnosed more than 10 years earlier had higher underuse and lower overuse than average. Finally, foreigners had higher underuse and higher overuse than did Italians.

The intraclass correlation for the three-level nested model for IC underuse was 0.10 at the district level and 0.25 at the GP within-district level. For IC overuse model, the ICC was 0.05 and 0.12, respectively.

Most of the ineligible patients placed in the IC plan had only one criterion for suspension, and only one patient met all criteria (Table 3A). The criterion for suspension most frequently found in patients overusing the IC plan was renal damage, followed by hospitalization for diabetes-related complications in the previous three years and by high HbA1c (Table 3B).

**Table 3:** Suspension criteria.

**a.**		
**N° of suspension criteria**	**N° of patients**	**%**
Only 1 criterion	1,563	90.9
2 criteria	149	8.7
3 criteria	7	0.4
4 criteria	1	0.1
**Total:**	1720	100.0
**b.**		
**Criteria**	**N° of patients**	**P**
eFGR	1123	*0*.*65*
At least 1 hosp.	409	*0*.*24*
HbA1c> = 9	241	*0*.*14*
Rapid insulin	113	*0*.*07*

Distribution by number (a.) and probability (P) (b.) of each met suspension criterion from the IC plan. Notes: the sum of probabilities (P) calculated as n° of patients with a specific suspension criterion/total number of patients with suspension criteria, exceeds 100% because many patients had more than one suspension criterion.

## Discussion

After several years since the introduction of the integrated care model, we found that only one-third of patients are managed in the integrated care plan. Three figures contribute to the proportion of integrated care plan use: only 37% of T2D patients fulfil the eligibility criteria, 37% of the eligible patients are not yet included, 15% of T2D patients are not included in any care plan. The strongest predictor of IC plan underuse is the district of residence and thus presumably due to healthcare provider factors: GPs’ and diabetes clinics’ attitude and organizational factors of the facilities, such as the time of implementation of the IC plan. The districts where underuse is lowest (i.e., the country districts of plain) are those with earliest IC care model introduction, while in the urban district (capital including the city of Reggio Emilia), where underuse is highest, there are still non-participating GPs. In districts with early IC care model introduction, the number of patients initially eligible to IC plan that lost eligibility criteria for aging or worsening of the disease could be higher than in the other districts. Moreover, the GP-patient relationship may differ based on the context (rural vs urban); a rural setting may facilitate the trust a patient has in disease management in primary care. Other factors have a small impact on underuse. Also, overuse is mostly linked to the district of residence. Nevertheless, the intraclass correlation measurements showed a weak clustering among districts and GPs, although the latter was slightly higher than the former in the two models.

Finally, the T2D patients neither in IC nor in DC plan were more likely to be residents in the urban district, to be older and to be recently diagnosed.

In a previous study, where the T2D patients were grouped in the same way, a higher percentage of patients neither in IC nor in DC plan (i.e., other-group) was found (36% vs. 15%), and consequently the other two were lower (IC: 27% vs. 33%; DC: 37% vs. 52%)[23]. Likewise, older patients were more likely to be part of other-group. In another study conducted in Turin [24], the percentage of patients seen by diabetes clinic (equivalent to our DC + IC) was lower (54% vs. our DC+IC = 85%). Once again, older patients were more likely to be part of other-group (called GP in the Turin study). Neither of these studies analyzed the appropriateness of adopted care plan based on eligibility criteria, focussing instead on adherence to recommended guidelines for monitoring diabetes.

Overall, the proportion of IC plan underuse is relatively high, but cannot be considered as inappropriate allocation per se. In fact, the inclusion in the integrated care plan is voluntary for both patients and GPs. Furthermore, as our ability to assess complications other than renal failure is limited by the available data sources, we probably overestimated the eligible population. This point is critical because, according to our findings, the inclusion criteria proposed by the Italian guidelines would limit the impact of the integrated model; even in the hypothesis of 100% appropriateness, only about one-third of patients could be allocated to the IC plan. Considering that, according to the management protocol, these patients need a follow-up visit at the diabetes clinic every two years, the IC plan does not dramatically decrease the diabetes clinic workload. Only less stringent eligibility criteria for IC plan could increase the impact of this care model.

On the other hand, a proportion of T2D patients are allocated to the IC plan even though they meet suspension criteria. By far the most disregarded criterion is the presence of renal failure. From a clinical point of view, this criterion is particularly important because it requires immediate re-evaluation of oral hypoglycaemic drug therapy [12]. Indeed, in some cases, these signs can be related to acute kidney injury rather than to chronic renal disease and the GP may have managed and resolved the acute episode, not considering this a sufficient criterion to move the patient definitively to diabetes clinic plan [25]. Despite this, it is now well recognized that while the glomerular filtration rate generally improves after acute kidney injury, the renal recovery process is often incomplete and can result in a chronic decrease in kidney function [26]. In this way, acute kidney injury increases the risk of advanced chronic kidney disease by more than threefold, independently of other risk factors of progression. Thus, even mild acute kidney injury in diabetic patients with relatively preserved renal function should be viewed as a serious event deserving a specialized evaluation [25].

Moreover, only specialized physicians can prescribe oral hypoglycaemic drug-classes useful for diabetic patients with chronic renal failure, such as iDPP4 or GLp1-analogs or Gliflozine. Despite this, the characteristics of these drugs may make GPs more comfortable not to formally change the care plan for these patients, who only need an annual therapeutic plan by diabetologists more than patients using metformin.

The most concerning finding in our study is the very high proportion of T2D patients without HbA1c and renal function test results among those patients who are not included in any formal care plan, compared to a previous study [24]. As for glycaemic control, the assessment of renal function in individuals with T2D is also extremely important since diabetic nephropathy constitutes a major cause of chronic kidney disease in the world, which makes diabetes the most frequent cause of end-stage renal disease [27,28].

Approximately 40% of all diabetic patients develop diabetic nephropathy [29], which is the most common diagnosis among individuals in renal replacement programmes, accounting for up to 44% of cases [30].The patients not included in any formal care plan represent a very heterogeneous group, which includes recently diagnosed, not yet evaluated people who explicitly do not want to be cared for by the public service, people who completely rely on their GP’s care and difficult-to-reach subgroups. It is possible that some of these patients undergo laboratory tests in completely private settings that do not report data to the registry, but it is also likely that some of these patients are actually underserved and not cared for. This hypothesis is consistent with the worse prognosis observed in this group in a previous study on the same population compared to patients in the IC or DC plan [16]. Therefore, the effort to reduce the number of patients not included in any formal care plan should be a priority.

### Strengths and limitations

This study assessed the adherence to the integrated care model, a chronic care approach for diabetes that attempts to allocate patients to the appropriate setting and to reduce the workload for the more specialized clinics. As this aim is common to many other chronic care models, our methodology and results could be useful in similar contexts to evaluate their performance or to plan monitoring indicators. Our study identifies patients with diabetes through a population-based registry that has a validated algorithm of case detection and is manually checked for the accuracy of information. The nature of this data source allowed us to classify patients into three groups, including those patients not in any defined care plan (i.e., in Other group). Our data source does not capture the test results from private laboratories. However, considering the coverage provided by the Italian National Health System in case of chronic diseases, the proportion of exams performed in private labs should be negligible. Furthermore, information about comorbidities that could contribute to choosing the most suitable plan was not completely available. Finally, we do not know what patients’ preferences are for a care plan.

In conclusion, patients are more often allocated to more a specialized setting than not: two-thirds of eligible patients are included in the integrated care plan while one-sixth of patients included in the integrated care plan are not eligible for this less specialised care setting. Healthcare provider-related factors are the most important determinants of over- and underuse of the integrated care plan.

## Supporting information

S1 Table Eligibility criteria. Eligibility criteria for integrated care plan of type 2 diabetes according to the Emilia-Romagna region guidelines [15].
(PDF)

S2 Table ICD-IX codes. ICD-IX codes for diabetes-related diagnosis.
(PDF)
