# Correction: Determinants of inappropriate setting allocation in the care of patients with type 2 diabetes: A population-based study in Reggio Emilia province

**DOI:** 10.1371/journal.pone.0221443

**Published:** 2019-08-15

**Authors:** Paola Ballotari, Francesco Venturelli, Valeria Manicardi, Massimo Vicentini, Francesca Ferrari, Marina Greci, Mariarosa Maiorana, Paolo Giorgi Rossi

An additional affiliation is missing for the seventh author. Mariarosa Maiorana is also affiliated with Clinical and Experimental Medicine PhD Program, University of Modena and Reggio Emilia, Modena, Italy.

## References

[1] Ballotari P, Venturelli F, Manicardi V, Vicentini M, Ferrari F, Greci M, et al (2019) Determinants of inappropriate setting allocation in the care of patients with type 2 diabetes: A population-based study in Reggio Emilia province. PLoS ONE 14(7): e0219965 10.1371/journal.pone.0219965 31329611 PMC6645528

