# Supplementary material for: Determinants of inappropriate setting allocation in the care of patients with type 2 diabetes: A population-based study in Reggio Emilia province
Source: PLoS One. 2019 Jul 22;14(7):e0219965. doi: 10.1371/journal.pone.0219965 (PMC6645528; doi:10.1371/journal.pone.0219965)
Supplement: S1 Table — Eligibility criteria for integrated care plan of type 2 diabetes according to the Emilia-Romagna region guidelines [15]. (PDF) [file pone.0219965.s001.pdf]

S1 Table Eligibility criteria

HEALTH CONDITION	INDICATOR	ALGORITHM	SOURCES	NOTES
having HbA1c* on target	HbA1c	last 2015 HbA1c level <=7% (53 mmol/mol) if aged less than 75 or <=8% (64 mmol/mol) if aged equal or more than 75	biochemistry laboratory database	if the measurement was unavailable, the subject was defined as unclassifiable
not being rapid-acting insulin user	drugs used other than ATC code = A10AB*	no rapid-acting insulin prescribed in 2015	drug prescription database	
not having mild or severe diabetes-related complications	hospital admission principal diagnosis	no hospital admission in the 2012-2014 period for diabetes-related causes^	hospital discharge database	
not having mild or severe renal complications	GFR~	last 2014-2015 GFR level>=60 ml/min/1.73m2	biochemistry laboratory database	if the measurement was unavailable, the subject was defined as unclassifiable

Eligibility criteria for integrated care plan of type 2 diabetes according to the Emilia-Romagna region guidelines [15]. *HbA1c =glycated haemoglobin. ^ see S2 Table: ICDIX codes for diabetes-related diagnosis. ~ GFR=glomerular filtration rate. When not available, we estimated glomerular filtration rate by using serum creatinine and Modification of Diet in Renal Disease (MDRD) Study equation [30]
