# Supplementary material for: Determinants of inappropriate setting allocation in the care of patients with type 2 diabetes: A population-based study in Reggio Emilia province
Source: PLoS One. 2019 Jul 22;14(7):e0219965. doi: 10.1371/journal.pone.0219965 (PMC6645528; doi:10.1371/journal.pone.0219965)
Supplement: S2 Table — ICD-IX codes for diabetes-related diagnosis. (PDF) [file pone.0219965.s002.pdf]

S2 Table ICD-IX codes

ICD-IX	Description
250.40	Diabetes with renal manifestations, type II or unspecified type, not stated as uncontrolled
250.42	Diabetes with renal manifestations, type II or unspecified type, uncontrolled
250.50	Diabetes with ophthalmic manifestations, type II or unspecified type, not stated as uncontrolled
250.52	Diabetes with ophthalmic manifestations, type II or unspecified type, uncontrolled
250.60	Diabetes with neurological manifestations, type II or unspecified type, not stated as uncontrolled
250.62	Diabetes with neurological manifestations, type II or unspecified type, uncontrolled
250.70	Diabetes with peripheral circulatory disorders, type II or unspecified type, not stated as uncontrolled
250.72	Diabetes with peripheral circulatory disorders, type II or unspecified type, uncontrolled
250.80	Diabetes with other specified manifestations, type II or unspecified type, not stated as uncontrolled
250.82	Diabetes with other specified manifestations, type II or unspecified type, uncontrolled
250.90	Diabetes with unspecified complication, type II or unspecified type, not stated as uncontrolled
250.92	Diabetes with unspecified complication, type II or unspecified type, uncontrolled
362.0*	Diabetic retinopathy
369*	Blindness and low vision
410*	Acute myocardial infarction
413*	Angina pectoris
414*	Other forms of chronic ischemic heart disease (excluded 414.1 - Aneurysm and dissection of heart)
430	Subarachnoid haemorrhage
431	Intracerebral haemorrhage
432*	Other and unspecified intracranial haemorrhage
434*	Occlusion of cerebral arteries
435.3	Vertebrobasilar artery syndrome
435.8	Other specified transient cerebral ischemias
435.9	Unspecified transient cerebral ischemia
436*	Acute, but ill-defined, cerebrovascular disease
437.1	Other generalized ischemic cerebrovascular disease
440.21; 440.22; 443.9	Claudication
583*	Nephritis and nephropathy not specified as acute or chronic
585*	Chronic kidney disease (CKD)
707.9	Chronic ulcer of unspecified site
V45.1; V56.0	Renal dialysis status; Extracorporeal dialysis
V49.70-V49.76; 785.4	Lower limb amputation status; gangrene

ICD-IX codes for diabetes-related diagnosis.
